# Purified anti-CD3 × anti-HER2 bispecific antibody potentiates cytokine-induced killer cells of poor spontaneous cytotoxicity against breast cancer cells

**DOI:** 10.1186/2045-3701-4-70

**Published:** 2014-11-25

**Authors:** Qingzhong He, Haisong Zhang, Youzhao Wang, Hong Hoi Ting, Wenhua Yu, Xuetao Cao, Wei Ge

**Affiliations:** National Key Laboratory of Medical Molecular Biology & Department of Immunology, Institute of Basic Medical Sciences, Chinese Academy of Medical Sciences, Dongdan Santiao 5 #, Dongcheng district, Beijing, 100005 China; Affiliated Hospital of Hebei University, No.212, Yu Hua East Rd, Nan Shi District, Baoding, Hebei 071000 China; JiangSu Laitai Medical Biotechnology Co., LTD, 3F, Building4, No.879 Zhongjiang Road, Shanghai, China; National Key Laboratory of Medical Molecular Biology & Department of Biochemistry and Molecular Biology, Institute of Basic Medical Sciences, Chinese Academy of Medical Sciences, Dongdan Santiao 5 #, Dongcheng district, Beijing, 100005 China

**Keywords:** Bispecific antibody, CIK cells, Chemical crosslinking, Protein purification

## Abstract

**Background:**

Chemical crosslinking is the most straightforward method to produce bispecific antibodies (BsAb) for arming *ex vivo* activated cytotoxic T lymphocytes. However, heterogeneous polymers are produced by chemical crosslinking. Currently, it is not known under what circumstances or to what extent further purification is needed.

**Results:**

In this study, we purified Traut’s Reagent-Sulfo-SMCC crosslinked anti-CD3 × anti-HER2 by size-exclusion column chromatography and compared the capacity of the crude and the purified forms of the BsAb in enhancing cytokine-induced killer (CIK) cell-mediated cytotoxicity *in vitro*. We found that the purified BsAb assisted CIK cells more efficiently than the crude form only when the spontaneous cytotoxicity of the CIK cells was relatively low; otherwise, the two forms performed almost identically.

**Conclusions:**

For the CIK cells of low spontaneous cytotoxicity, purified BsAb is a more powerful substitute for crude BsAb in enhancing their killing efficacy. However, that purification of BsAb is not necessary for robust CIK cells. This phenomenon also corroborates that CIK-mediated cytotoxicity is highly dependent on cell contact.

**Electronic supplementary material:**

The online version of this article (doi:10.1186/2045-3701-4-70) contains supplementary material, which is available to authorized users.

## Background

The concept of using hybrid antibodies to direct the cytotoxicity of effector cells to their intended target emerged in the 1980s [1]. The hybrid antibodies, produced by chemical crosslinking [2, 3] or hybrid hybridomas [4, 5] possess at least two different Fab fragments simultaneously, thus bridging effector and target cells by antibody-antigen recognition. Consequently, these are also called bispecific antibodies (BsAbs).

In 1990, Nitta et al. first combined cytokine-induced immune effector cells and BsAbs to treat malignant glioma. Compared with untreated lymphokine-activated killer (LAK) cells, BsAb-armed LAK cells efficiently eradicated glioma cells and prolonged patients’ lifespan. Subsequently, several groups applied BsAbs to direct various versions of LAK cells towards ovarian carcinoma [6], colon carcinoma [7], small-cell lung carcinoma [8], breast cancer [9] or B-lymphoma [10]. Some of these therapies have entered clinical trials [11].

In past decades, novel methods have been developed to produce BsAbs, including diabodies [12], and duobodies [13]. However, direct chemical crosslinking of two monoantibody populations, which involves the use of existing antibodies and does not involve additional bioengineering, is still the most straightforward and widely used method [14–17]. Although there were once a concern for recruiting Fcγ receptor cells [18], they are tolerable at the dose used to arm LAK-like cells and experience of the trifunctional antibody drug Ertumaxomab indicates a potential benefit [19].

Because site-specific conjugation methods are unavailable, the product of chemical crosslinking is always heterogeneous, with more than 50% of the total protein mass of the product reported to comprise IgG monomers [9]. These will occupy binding sites but not bridge effector and target cells. Therefore, theoretically when the conjugation product is applied as a whole, the existence of monomers will interfere with the performance of IgG hetero-polymers. But considering that the number of binding sites on cells and the threshold of bridge number to trigger cytotoxicity also influence the performance of the chemical conjugate, we need to assess to what extend the monomers influence the performance and whether further purification of the chemical conjugate is necessary or not.

Here, we crosslinked anti-CD3 and anti-HER2 chemically and purified the crude conjugation product with size-exclusion chromatography to an IgG dimer purity of >70%. The potency of the crude and the purified BsAb was then assessed in directing the cytotoxicity of cytokine-induced killer (CIK) cells [20] against human SK-BR-3 breast cancer cells. The purified BsAb potentiated CIK cells of poor spontaneous cytotoxicity more efficiently than the crude form, while their performance was indistinguishable when applied to CIK cells of robust spontaneous cytotoxicity. These results suggest that further purification to remove IgG monomers from chemically conjugated BsAb is always unnecessary; however, when spontaneous cytotoxicity of CIK cells is relatively weak, purified BsAb may enhance the performance more efficiently. Our findings also indicate that the mechanism by which CIK cells kill tumor cells is highly dependent on cell membrane adherent molecules; thus, variation in the spontaneous cytotoxicity of CIK cells among different donors or culture times is influenced by the expression of these molecules.

## Results

### Traut’s reagent and sulfo-SMCC efficiently crosslink anti-CD3 and anti-HER2

The sulfhydryl-mediated reaction was employed to crosslink anti-CD3 and anti-HER2. For maximal crosslinking efficiency, several crosslinker pairs were screened.

**Figure 1:**
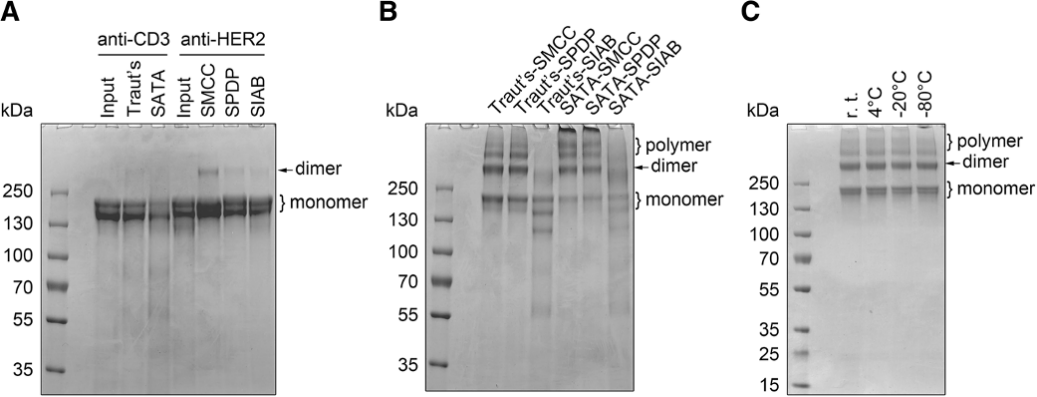
**Chemical crosslinking of anti**-**CD3 and anti-** **HER2.** **(A)** Native electrophoresis of crosslinker-conjugated IgG monomer. Input represents unconjugated control. 10 μg protein per lane. **(B)** Native electrophoresis of crosslinked anti-CD3 × anti-HER2 with combinations of crosslinkers. 10 μg protein per lane. **(C)** Native electrophoresis of Traut’s-SMCC-crosslinked anti-CD3 × anti-HER2 following storage under various conditions for 3 months (r. t, room temperature). 10 μg protein per lane.

Traut’s Reagent or SATA were conjugated with anti-CD3 to introduce sulfhydryls, while Sulfo-SMCC, Sulfo-LC-SPDP or Sulfo-SIAB were conjugated with anti-HER2 to introduce functional groups that react with sulfhydryls. After conjugation, no molecular weight shift of the monomer band or obvious polymerization was observed in the product (Figure 1A). A faint band with a migration pattern corresponding to that of an IgG dimer was detected in anti-HER2 samples, but was shown to represent less than 1% of the total protein by densitometry (Figure 1A).

After conjugation, equal molar quantities of sulfhydryl-containing anti-CD3 and sulfhydryl-reacting anti-HER2 were mixed with all combinations of the crosslinkers and the crosslinking products were resolved by native protein electrophoresis. Because crosslinkers reacted with IgG in 5-fold or more molar excess, each IgG molecule was linked with multiple sulfhydryls or sulfhydryl-reacting functional groups. As expected, heteroconjugation of anti-CD3 × anti-HER2 produced a series of molecules including monomers, dimers, and trimers (Figure 1B).

Anti-CD3 × anti-HER2 BsAb is designed to mediate contact between cytotoxic T cells and tumor cells expressing high levels of HER2; thus, anti-CD3 × anti-HER2 dimers are the ideal effectors. Among the combinations of crosslinkers, Traut’s-SMCC produced the largest proportion of dimers (Figure 1B). The dimer yield of Traut’s-SPDP was comparable to that of Traut’s-SMCC (Figure 1B); however, crosslinking involving SPDP requires an overnight incubation, which is much longer than that required for SMCC. Products of SATA crosslinking contained less monomers but more insoluble aggregates than those of Traut’s Reagent, which indicated over-crosslinking. Unexpectedly, bands of aberrant molecular weight were detected among the products of Sulfo-SIAB crosslinking, which might be a consequence of the reagent itself.

Based on the crosslinking efficiency, the combination of Traut’s Reagent and Sulfo-SMCC was used for BsAb production in the following experiments.

Traut’s-SMCC-conjugated BsAb was stable during a 3-month storage at room temperature, 4°C, -20°C or -80°C (Figure 1C).

### Size-exclusion chromatography purified the crude conjugation product to a dimer purity of >70%

**Figure 2:**
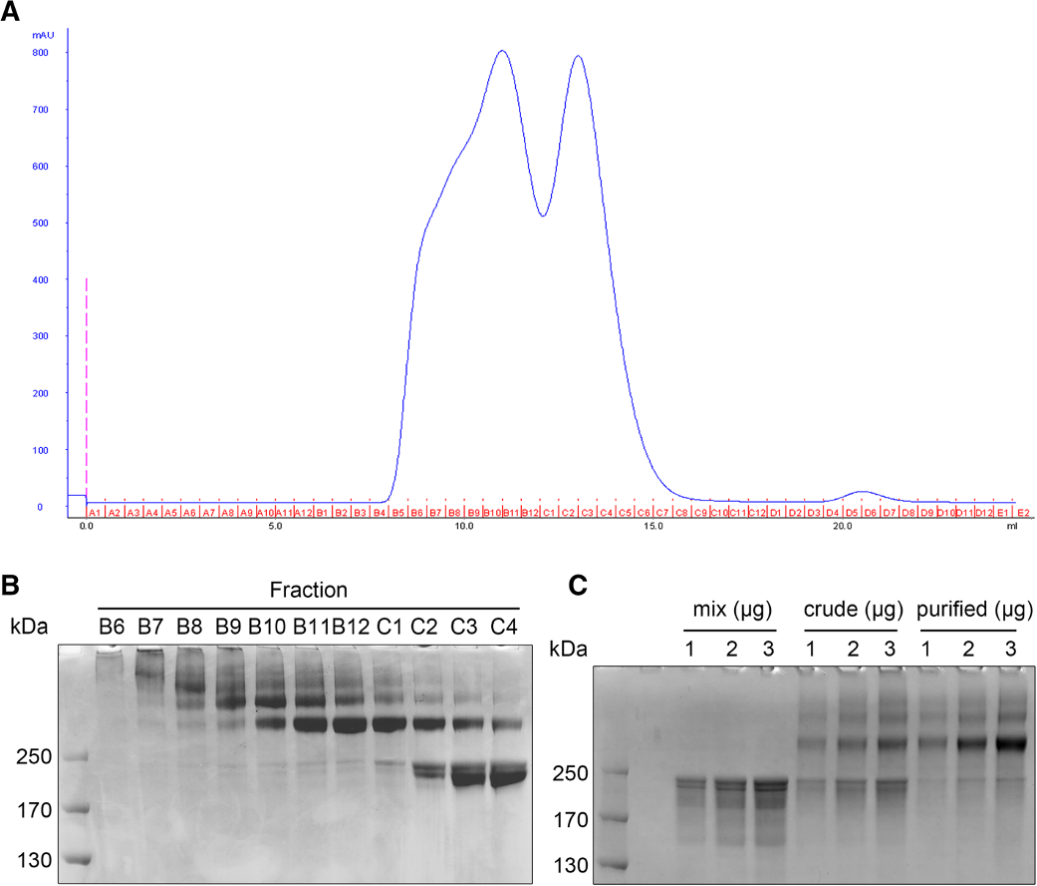
**Purification of crude BsAb by size**-**exclusion column chromatography.** **(A)** UV profile of Traut’s-SMCC-crosslinked BsAb running in Superdex 200 10/300 GL. **(B)** Native electrophoresis of Fractions B6 to C4. Equal proportions (2%) of each fraction were loaded in each well. **(C)** Native electrophoresis of anti-CD3/anti-HER2 equal molar mixture (mix), crude crosslinking product (crude) and purified BsAb (purified).

As shown in Figure 1B, IgG monomer comprised approximately 40% of the crosslinking product. The monomer interferes with anti-CD3 × anti-HER2 dimer bridging of CIK cells and tumor cells by occupying CD3 or HER2 on the cell membrane. To maximize the efficacy of the product, IgG monomer was removed by size-exclusion chromatography. In resolution using a Superdex 200 10/300 GL column, the monomer was well separated from dimer and polymer (Figure 2A).

Gel filtration fractions were evaluated by native electrophoresis (Figure 2B), and the pooled Fractions B11, B12 and C1 were designated as “purified BsAb”. As shown in Figure 2C, the dimer proportion in the purified product, which reached >70% according to densitometric analysis, was approximately twice that in the crude product. However, the yield of purified BsAb was only approximately 15%.

Bio-Rad gel filtration standards were separated on the same column to determine the molecular weight of the IgG dimer and monomer. The molecular weight and the elution volume of each peak were recorded to establish a standard curve (Additional file 1: Figure S1). Using the formula of the standard curve, the molecular weight of the monomer was calculated as 130.60 kDa (Additional file 1: Table S3), which was slightly smaller than the expectation of 150 kDa; while that of the dimer was 378.48 kDa (Additional file 1: Table S3), which was larger than the expectation of 261.20 kDa but very close to that of the trimer (391.80 kDa). It is impossible for the crosslinking reaction to form a trimer without forming a dimer; thus, we did not consider that the peak corresponded to a trimer but instead, speculated that the shift resulted from an extended molecular shape.

### Purified BsAb assisted the CIK cells of poor spontaneous cytotoxicity more efficiently than did crude BsAb

To evaluate the efficacy of purified BsAb in enhancing CIK cell cytotoxicity, *in vitro* cytotoxicity assays were carried out using the SK-BR-3 breast cancer cell line as targets. A single batch of CIK cells in continuous culture was used in all the assays described.

**Figure 3:**
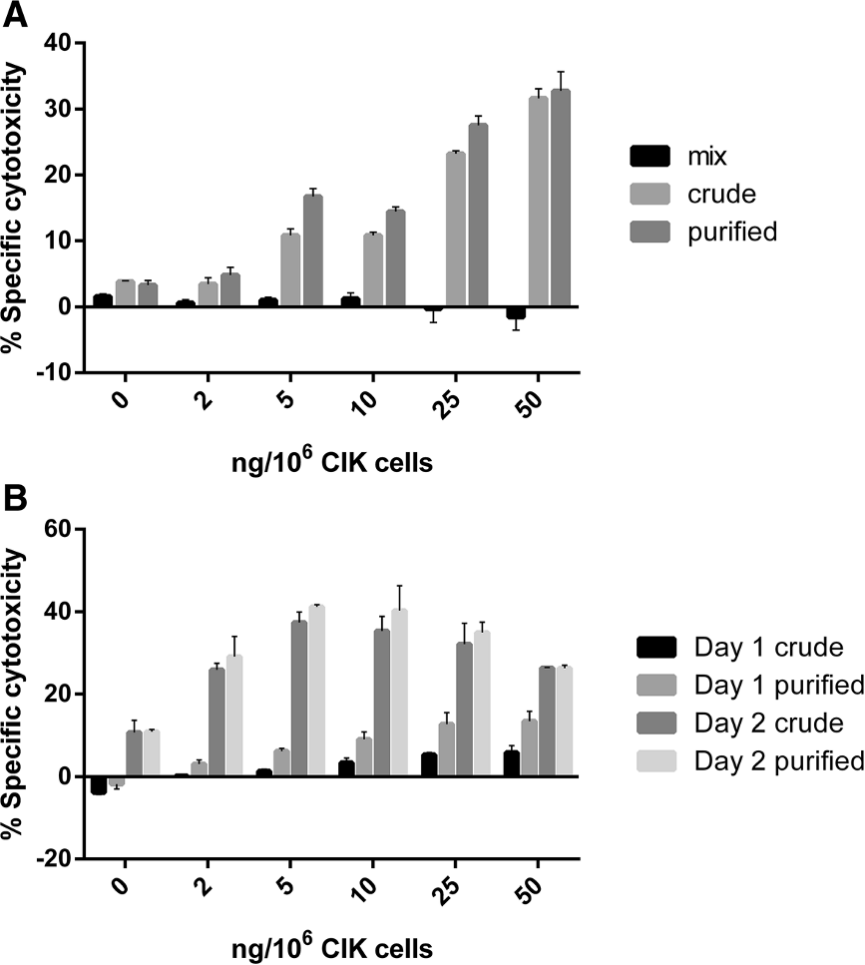
**BsAb**-**armed CIK**-**mediated cytotoxicity against SK**-**BR**-**3.** **(A)** Specific cytotoxicity mediated by CIK cells armed with 0–50 ng antibody per 10^6^ cells (mix, anti-CD3/anti-HER2 equal molar mixture). **(B)** Specific cytotoxicity of the same batch of CIK cells on two consecutive days. The results shown in the figures were representative of at least two independent experiments.

As shown in Figure 3A, the mixture of anti-CD3 and anti-HER2 did not direct CIK cell cytotoxicity against SK-BR-3 cells, while both crude and purified BsAb enhanced the cytotoxicity against these targets. When less than 50 ng of BsAb per 10^6^ effector cells was applied in an effector cell vs. target cell (E:T) ratio of 5, BsAb assisted CIK cell cytotoxicity in a dose-dependent effect. However, no significant difference was observed between the effects mediated by purified and crude BsAb.

It is well-recognized that the spontaneous cytotoxicity of a single batch of CIK cells in continuous culture varies on a daily basis [9], and that the cytotoxicity of CIK cells varies from donor to donor [21]. In further studies, we found that the spontaneous cytotoxicity of CIK cells varied between the crude and the purified BsAb. As shown in Figure 3B, assays performed with the CIK cells on two consecutive days revealed that, when the spontaneous cytotoxicity was negligible, the CIK cells armed with purified BsAb exhibited at least two-fold cytotoxicity compared with that when armed with crude BsAb (*t*-test: p < 0.05); however, on the second day when the spontaneous cytotoxicity was as high as 10%, there was no significant difference between the effects of the purified and crude BsAb (*t*-test: p > 0.05). Furthermore, we found that spontaneous cytotoxicity was also associated with the saturating amount of BsAb. When the spontaneous cytotoxicity of CIK was approximately 10%, 5 ng BsAb was enough to saturate 10^6^ CIK cells. However, when the spontaneous cytotoxicity was low, at least 25 ng BsAb was required for saturation.

We also tested the stability of the purified BsAb following lyophilization. Purified BsAb in PBS was directly lyophilized and reconstituted with ddH_2_O. The lyophilization-reconstitution cycle caused approximately 10% soluble protein loss (data not shown); however, the efficacy of reconstituted BsAb was slightly higher than that of the original sample (Additional file 1: Figure S2). This may be because polymers are more prone to precipitation following lyophilization than oligomers.

## Discussion

Chemical crosslinking is the most convenient and rapid method to produce BsAb. However, the product of chemical crosslinking comprises a heterogeneous series of conjugates, including IgG monomers, heterodimers, heterotrimers and so on. The mixture of anti-CD3 and anti-HER2 did not enhance CIK cytotoxicity cell against SK-BR-3 (Figure 3A). Furthermore, the dose of BsAb used in cell-mediated cytotoxicity assays is non-toxic to tumor cells (Additional file 1: Figure S3). Therefore, the only function of BsAb should be establishing contact between the effector and target cells. A heterodimer may mediate contact between two CIK cells and two tumor cells at most. In contrast, monomers affect the function of dimers by occupying cell surface molecules and conjugates of three or more IgGs may lead to ineffective contact between the same cell populations. In this report, we purified chemically conjugated BsAb and elevated the purity of the heterodimer to more than 70%.

We predicted that the purified BsAb would enhance CIK cell-mediated cytotoxicity by two-fold compared with that of the crude form. However, our results demonstrated that the efficacy of the purified BsAb was associated with the capacity for spontaneous cytotoxicity of CIK cells. When the spontaneous cytotoxicity of CIK cells was undetectable, the purified BsAb potentiated the effects of CIK cells much more efficiently than did the crude form; however, when the spontaneous cytotoxicity of CIK cells was as high as 10%, the purified and the crude BsAb performed almost identically. This phenomenon indicates that CIK cell-mediated cytotoxicity to tumor cells is highly dependent on cell contact.

It has long been observed that the cytotoxicity of a single batch of CIK cells in continuous culture varies day by day [9]. CIK cells are heterogeneous. The major cytotoxic population of CIK cells comprises CD3^+^CD56^+^ cells [22–24]. As the cytotoxicity assays were performed in consecutive two days (Figure 3B), the variation in the spontaneous cytotoxicity was unlikely to be due to the propagation of cytotoxic cells but is more likely to be affected by the expression of certain cell surface adherent molecules. Based on this assumption, we propose that cells with robust capacity for spontaneous cytotoxicity possess abundant adherent molecules, thus, allowing for saturated contact between CIK cells and tumor cells. This would account for the observation that the purified and crude forms of BsAb performed almost identically. However, when the capacity for spontaneous cytotoxicity of cells is undetectable, indicating that the cells possess few adherent molecules, the contact between CIK cells and tumor cells is far from being saturated. Thus, when applied in the same dose, the purified BsAb mediated more CD3-HER2 bridges than the crude, which accounts for the greater potentiation of CIK cell cytotoxicity mediated by the purified BsAb compared with that mediated by the crude product.

In summary, our study demonstrates that the purified chemically conjugated BsAb assists the CIK cells of poor spontaneous cytotoxicity more efficiently than the crude, which implicates that for the cancer patients whose autologous CIK cells are of low spontaneous cytotoxicity, the purified BsAb is a more powerful arming reagent substitute for the crude BsAb. If the spontaneous cytotoxicity of CIK cells is relatively high, purification of chemically conjugated BsAb is not necessary.

## Material and methods

### Cells

CIK cells were prepared as described in Schmit-Worf et al. [20] with slight modifications. Briefly, lymphocytes were isolated from peripheral blood obtained from cancer patients by Ficoll gradient centrifugation. At Day 0, non-adherent lymphocytes were adjusted to a final concentration of 1–3 × 10^6^ cells/mL and stimulated with 300–500 U/mL human interleukin-4 (IL-4) and 1,000 U/mL interferon-γ (IFN-γ) for 24 h. Then cells were treated with 50–100 ng/mL soluble anti-CD3 (TONBO Biosciences, San Diego, CA, US, clone: OKT3) and 100 U/mL human recombinant interleukin-1α (rIL-1α) for 48 h. Medium supplemented with recombinant interleukin-2 (rIL-2) was refreshed every three days. Experiments were carried out with these CIK cells at approximately Day 14.

The human breast cancer cell line SK-BR-3 was purchased from the Cell Resource Center, Institute of Basic Medical Sciences, Chinese Academy of Medical Sciences.

### Antibody chemical crosslinking

Mouse monoclonal anti-CD3 antibody was purchased from TONBO Biosciences (clone: OKT3, REF: 40-0037-U500).

Humanized anti-HER2 monoantibody was purchased from Roche (trastuzumab/Herceptin®).

Traut’s Reagent (2-Iminothiolane · HCl), N-Succinimidyl S-acetylthioacetate (SATA), sulfosuccinimidyl 4-[N-maleimidomethyl]cyclohexane-1-carboxylate (Sulfo-SMCC), sulfosuccinimidyl 6-[3’(2-pyridyldithio)-propionamido] hexanoate (Sulfo-LC-SPDP) and sulfosuccinimidyl[4-iodoacetyl]aminobenzoate (Sulfo-SIAB) were purchased from Pierce Biotechnology, Thermo SCIENTIFIC.

Buffer changing and protein desalting was performed with Zeba Spin Desalting Columns (MWCO: 7 kDa, Thermo SCIENTIFIC). IgG was concentrated with Amicon Ultra Centrifugal Filters. Crosslinking reactions were performed on an Eppendorf Thermomixer at 300 rpm.

Chemical crosslinking of antibodies was carried out following the manufacturer’s instructions provided with the crosslinking reagents. Detailed conditions for the conjugation of crosslinker and IgG are shown in Additional file 1: Table S1. Conditions for heterocrosslinking of two conjugated IgGs are shown in Additional file 1: Table S2.

To evaluate BsAb in native electrophoresis, samples were mixed with one third volume of 4× NuPAGE LDS Sample Buffer (Novex, Life Technologies), electrophoresed in a NuPAGE 4–12% Bis-Tris Gel (Novex, Life Technologies) and visualized with Commassie brilliant blue staining.

### Size-exclusion chromatography

Crude BsAb (6 mg) was loaded onto a Superdex 200 10/300 GL gel filtration column (GE Healthcare) attached to the ÄKTApurifier (GE). PBS (Thermo SCIENTIFIC, #1890535) was used as running buffer. Each fraction comprised 0.5 mL.

For molecular weight calculations, a gel filtration standard (BIO-RAD, #151-1901) was loaded onto the same column and run under the same conditions. The molecular weight and the corresponding elution volume of peaks was recorded to formulate a standard curve using the regression analysis function of Microsoft Excel 2013. The molecular weight of BsAb was estimated from the standard curve.

### Cytotoxicity assay

*In vitro* cytotoxicity assays were carried out with the CytoTox 96® Non-Radioactive (LDH) Cytotoxicity Assay Kit (Promega #G1780) following the manufacturer’s instructions.

To determine cell-mediated cytotoxicity, CIK cells were suspended in modified RPMI-1640 medium (HyClone #SH30809.01B) containing 5% fetal bovine serum (FBS, Gibco #10099-141). The intended amount (0-50 ng per 10^6^ cells) of BsAb was added to the suspension, and 30 minutes later non-coupled BsAb was washed out with the same medium. BsAb-armed CIK cells (1 × 10^5^) and 2 × 10^4^ SK-BR-3 cells suspended in the medium were plated into each well of a 96-well plate. After a 6-hour incubation at 37°C, 50 μL supernatant from each well was used in the LDH assay. OD_490_ was measured with the Varioskan Flash Multimode Reader (Thermo SCIENTIFIC) to calculate CIK-mediated specific cytotoxicity towards SK-BR-3.

To determine antibody-mediated cytotoxicity, SK-BR-3 cells (2 × 10^4^ cells/well) were plated into 96-well plates. 50 ng of the anti-CD3/anti-HER2 mixture, crude or purified BsAb was added to the wells immediately. After a 6-hour incubation at 37°C, 50 μL supernatant from each well was used in the LDH assay. OD_490_ was measured to calculate cytotoxicity towards SK-BR-3.

### Densitometry

Densitometry of stained protein bands was performed with ImageJ software [25].

### Ethical approval

The protocol was approved by Ethics Committee of Institute of Basic Medical Sciences, Chinese Academy of Medical Sciences (reference number: CAMS012-2014).

## Electronic supplementary material

Additional file 1:**anti-CD3xanti-HER2 BsAb s1.**(DOCX 248 KB)

## Abbreviations

BsAb: Bispecific antibody
IgG: Immunoglobulin G
LAK: lymphokine-activated killer
CIK: Cytokine-induced killer
IL-4: Interleukin-4
IFN-γ: Interferon-γ
rIL-1α: Recombinant interleukin-1α
rIL-2: Recombinant interleukin-2
SATA: N-Succinimidyl S-acetylthioacetate
Sulfo-SMCC: Sulfosuccinimidyl 4-[N-maleimidomethyl]cyclohexane-1-carboxylate
Sulfo-LC-SPDP: Sulfosuccinimidyl 6-[3’(2-pyridyldithio)-propionamido] hexanoate
Sulfo-SIAB: Sulfosuccinimidyl [4-iodoacetyl] aminobenzoate
E: T: Effector cell: target cell
r. t: room temperature.

## References

[1] Karpovsky B, Titus JA, Stephany DA, Segal DM (1984). Production of target-specific effector cells using hetero-cross-linked aggregates containing anti-target cell and anti-Fc gamma receptor antibodies. J Exp Med.

[2] Perez P, Hoffman RW, Shaw S, Bluestone JA, Segal DM (1985). Specific targeting of cytotoxic T cells by anti-T3 linked to anti-target cell antibody. Nature.

[3] Staerz UD, Kanagawa O, Bevan MJ (1985). Hybrid antibodies can target sites for attack by T cells. Nature.

[4] Staerz UD, Bevan MJ (1986). Hybrid hybridoma producing a bispecific monoclonal antibody that can focus effector T-cell activity. Proc Natl Acad Sci U S A.

[5] Lanzavecchia A, Scheidegger D (1987). The use of hybrid hybridomas to target human cytotoxic T lymphocytes. Eur J Immunol.

[6] Moller SA, Reisfeld RA (1991). Bispecific-monoclonal-antibody-directed lysis of ovarian carcinoma cells by activated human T lymphocytes. Cancer Immunol Immunother.

[7] Kuppen PJ, Eggermont AM, Smits KM, van Eendenburg JD, Lazeroms SP, van de Velde CJ, Fleuren GJ (1993). The development and purification of a bispecific antibody for lymphokine-activated killer cell targeting against the rat colon carcinoma CC531. Cancer Immunol Immunother.

[8] Azuma A, Yagita H, Okumura K, Kudoh S, Niitani H (1994). Potentiation of long-term-cultured lymphokine-activated killer cell cytotoxicity against small-cell lung carcinoma by anti-CD3 x anti-(tumor-associated antigen) bispecific antibody. Cancer Immunol Immunother.

[9] Sen M, Wankowski DM, Garlie NK, Siebenlist RE, Van Epps D, LeFever AV, Lum LG (2001). Use of anti-CD3 x anti-HER2/neu bispecific antibody for redirecting cytotoxicity of activated T cells toward HER2/neu + tumors. J Hematother Stem Cell Res.

[10] Tita-Nwa F, Moldenhauer G, Herbst M, Kleist C, Ho AD, Kornacker M (2007). Cytokine-induced killer cells targeted by the novel bispecific antibody CD19xCD5 (HD37xT5.16) efficiently lyse B-lymphoma cells. Cancer Immunol Immunother.

[11] Lum LG, Rathore R, Cummings F, Colvin GA, Radie-Keane K, Maizel A, Quesenberry PJ, Elfenbein GJ (2003). Phase I/II study of treatment of stage IV breast cancer with OKT3 x trastuzumab-armed activated T cells. Clin Breast Cancer.

[12] Holliger P, Prospero T, Winter G (1993). “Diabodies”: small bivalent and bispecific antibody fragments. Proc Natl Acad Sci U S A.

[13] Labrijn AF, Meesters JI, de Goeij BE, van den Bremer ET, Neijssen J, van Kampen MD, Strumane K, Verploegen S, Kundu A, Gramer MJ, van Berkel PH, van de Winkel JG, Schuurman J, Parren PW (2013). Efficient generation of stable bispecific IgG1 by controlled Fab-arm exchange. Proc Natl Acad Sci U S A.

[14] Lum LG, Ramesh M, Thakur A, Mitra S, Deol A, Uberti JP, Pellett PE (2012). Targeting cytomegalovirus-infected cells using T cells armed with anti-CD3 x anti-CMV bispecific antibody. Biol Blood Marrow Transplant.

[15] Thakur A, Schalk D, Sarkar SH, Al-Khadimi Z, Sarkar FH, Lum LG (2012). A Th1 cytokine-enriched microenvironment enhances tumor killing by activated T cells armed with bispecific antibodies and inhibits the development of myeloid-derived suppressor cells. Cancer Immunol Immunother.

[16] Yankelevich M, Kondadasula SV, Thakur A, Buck S, Cheung NK, Lum LG (2012). Anti-CD3 x anti-GD2 bispecific antibody redirects T-cell cytolytic activity to neuroblastoma targets. Pediatr Blood Cancer.

[17] Huang J, Li C, Wang Y, Lv H, Guo Y, Dai H, Wicha MS, Chang AE, Li Q (2013). Cytokine-induced killer (CIK) cells bound with anti-CD3/anti-CD133 bispecific antibodies target CD133(high) cancer stem cells in vitro and in vivo. Clin Immunol.

[18] Mezzanzanica D, Canevari S, Menard S, Pupa SM, Tagliabue E, Lanzavecchia A, Colnaghi MI (1988). Human ovarian carcinoma lysis by cytotoxic T cells targeted by bispecific monoclonal antibodies: analysis of the antibody components. Int J Cancer.

[19] Kiewe P, Thiel E (2008). Ertumaxomab: a trifunctional antibody for breast cancer treatment. Expert Opin Investig Drugs.

[20] Schmidt-Wolf IG, Negrin RS, Kiem HP, Blume KG, Weissman IL (1991). Use of a SCID mouse/human lymphoma model to evaluate cytokine-induced killer cells with potent antitumor cell activity. J Exp Med.

[21] Linn YC, Lau LC, Hui KM (2002). Generation of cytokine-induced killer cells from leukaemic samples with in vitro cytotoxicity against autologous and allogeneic leukaemic blasts. Br J Haematol.

[22] Schmidt-Wolf IG, Lefterova P, Mehta BA, Fernandez LP, Huhn D, Blume KG, Weissman IL, Negrin RS (1993). Phenotypic characterization and identification of effector cells involved in tumor cell recognition of cytokine-induced killer cells. Exp Hematol.

[23] Lu PH, Negrin RS (1994). A novel population of expanded human CD3 + CD56+ cells derived from T cells with potent in vivo antitumor activity in mice with severe combined immunodeficiency. J Immunol.

[24] Schmidt-Wolf IG, Lefterova P, Johnston V, Huhn D, Blume KG, Negrin RS (1994). Propagation of large numbers of T cells with natural killer cell markers. Br J Haematol.

[25] Schneider CA, Rasband WS, Eliceiri KW (2012). NIH Image to ImageJ: 25 years of image analysis. Nat Methods.

