# Supplementary material for: Purified anti-CD3 × anti-HER2 bispecific antibody potentiates cytokine-induced killer cells of poor spontaneous cytotoxicity against breast cancer cells
Source: Cell Biosci. 2014 Nov 25;4:70. doi: 10.1186/2045-3701-4-70 (PMC4258008; doi:10.1186/2045-3701-4-70)
Supplement: Supplementary file 1 — Additional file 1:anti-CD3xanti-HER2 BsAb s1.(DOCX 248 KB) [file 13578_2014_194_MOESM1_ESM.docx]

| **Antibody** | **anti-CD3** | | **anti-HER2** | | |
| --- | --- | --- | --- | --- | --- |
| **Crosslinker** | **Traut's Reagent** | **SATA** | **Sulfo-SMCC** | **Sulfo-LC-SPDP** | **Sulfo-SIAB** |
| **Crosslinker Solvent** | H_2_O | DMSO | H_2_O | H_2_O | H_2_O |
| **Reaction Buffer** | 100 mM sodium phosphate, 150 mM sodium chloride, 2 mM EDTA, pH 8.0 | 100 mM sodium phosphate, 150 mM sodium chloride, pH 7.2 | 100 mM sodium phosphate, 150 mM sodium chloride, 2 mM EDTA, pH 7.2 | 100 mM sodium phosphate, 150 mM sodium chloride, 2 mM EDTA, pH 7.6 | 50 mM sodium borate, pH 8.5, 5 mM EDTA (reaction in dark) |
| **10× Deacetylation Solution** | \ | Reaction Buffer with 0.5 M hydroxylamine, 25 mM EDTA, pH 7.2 | \ | \ | \ |
| **Product Buffer A (for SMCC)** | 100 mM sodium phosphate, 150 mM sodium chloride, 2 mM EDTA, pH 7.2 | 100 mM sodium phosphate, 150 mM sodium chloride, 2 mM EDTA, pH 7.2 | 100 mM sodium phosphate, 150 mM sodium chloride, 2 mM EDTA, pH 7.2 | \ | \ |
| **Product Buffer B (for SPDP)** | 100 mM sodium phosphate, 150 mM sodium chloride, 2 mM EDTA, pH 7.6 | 100 mM sodium phosphate, 150 mM sodium chloride, 2 mM EDTA, pH 7.6 | \ | 100 mM sodium phosphate, 150 mM sodium chloride, 2 mM EDTA, pH 7.6 | \ |
| **Product Buffer C (for SIAB)** | 50 mM sodium borate, pH 8.5, 5 mM EDTA | 50 mM sodium borate, pH 8.5, 5 mM EDTA | \ | \ | 50 mM sodium borate, pH 8.5, 5 mM EDTA (product in dark) |
| **Molar Ratio (Protein:Crosslinker)** | 1:10 | 1:9 | 1:10 | 1:20 | 1:5 |
| **Reaction Temperature** | 25°C | 25°C | 25°C | 25°C | 25°C |
| **Reaction Duration** | 1 hr | 30 min (conjugation), 2 hr (deacetylation) | 1 hr | 30 min | 30 min (in dark) |

Supplemental Table 1 Detailed crosslinker conjugating condition and buffer components.

|  | | **Sulfo-SMCC** | **Sulfo-LC-SPDP** | **Sulfo-SIAB** |
| --- | --- | --- | --- | --- |
| **Conjugating** | **Reaction Buffer** | 100 mM sodium phosphate, 150 mM sodium chloride, 2 mM EDTA, pH 7.2 | 100 mM sodium phosphate, 150 mM sodium chloride, 2 mM EDTA, pH 7.6 | 50 mM sodium borate, pH 8.5, 5 mM EDTA (reaction in dark) |
|  | **Molar Ratio (Protein:Protein)** | 1:1 | 1:1 | 1:1 |
|  | **Reaction Temperature** | 25°C | 25°C | 25°C |
|  | **Reaction Duration** | 30 min | Overnight (8 hr) | 1 hr |
| **Quenching** | **10× Quenching Solution** | Not Required | Not Required | 50 mM cysteine, 50 mM sodium borate, pH 8.5, 5 mM EDTA (reaction in dark, desalting required post-quenching) |
|  | **Quenching Temperature** |  |  | 25°C |
|  | **Quenching Duration** |  |  | 15 min |

Supplemental Table 2 Detailed crosslinking condition of anti-CD3 and anti-HER2 bearing crosslinkers.


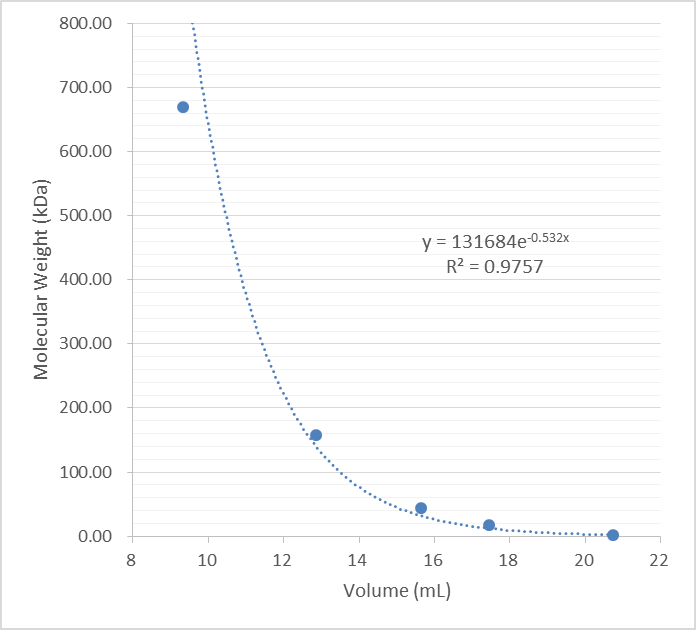


Supplemental Figure 1 Standard curve calculated from BIO-RAD Gel Filtration Standard.

|  | Molecule | Molecular Weight (kDa) | Volume (mL) |
| --- | --- | --- | --- |
| Gel Filtration Standard | Thyroglobulin (bovine) | 670.00 | 9.32 |
|  | γ-globulin (bovine) | 158.00 | 12.85 |
|  | Ovalbumin (chicken) | 44.00 | 15.64 |
|  | Myoglobin (horse) | 17.00 | 17.45 |
|  | Vitamin B_12_ | 1.35 | 20.73 |
| Sample | dimer | 378.48 | 11.00 |
|  | monomer | 130.60 | 13.00 |

Supplemental Table 3 Calculated molecular weight of BsAb dimer and IgG monomer.





Supplemental Figure 2 Specific cytotoxicity mediated by CIK cells armed with 5 ng original or reconstituted purified BsAb per 10^6^ cells. E:T=5:1.





Supplemental Figure 3 50-ng BsAb-mediated cytotoxicity towards 2×10^4^ SK-BR-3 cells.
